# Variation in renal biopsy and medication prescribing practices among pediatric Medicaid patients with lupus nephritis prior to end-stage renal disease in the US, 2000 to 2004

**DOI:** 10.1186/ar3992

**Published:** 2012-09-27

**Authors:** LT Hiraki, CH Feldman, J Liu, GS Alarcón, MA Fischer, WC Winkelmayer, KH Costenbader

**Affiliations:** 1 Harvard School of Public Health, Boston, MA, USA; 2 Brigham and Women's Hospital, Boston, MA, USA; 3 University of Alabama at Birmingham, Birmingham, AL, USA; 4 Stanford University School of Medicine, Palo Alto, CA, USA

## Background

Unequal medical care may contribute to striking sociodemographic disparities seen in outcomes for children with lupus nephritis. Medicaid is the US federal-state program providing health insurance to low-income children and parents. We investigated US nationwide variation in renal biopsies and medication prescriptions for children with lupus nephritis enrolled in Medicaid, 2000 to 2004, in the months preceding end-stage renal disease (ESRD).

## Methods

We identified all children aged 3 to <18 years with SLE (≥3 ICD-9 codes of 710.0, each >30 days apart) in the Medicaid Analytic eXtract (MAX) from 2000 to 2004, which contains outpatient and inpatient Medicaid claims for enrollees in 47 US states and the District of Columbia. These data were linked to the US Renal Data System, with information on essentially all ESRD patients in the US, for the same years. We compared frequencies of renal biopsies, and prescription of corticosteroids, hydroxychloroquine (HCQ) and immunosuppressants (mycophenylate mofetil (MMF), cyclophosphamide (CYC), cyclosporine, azathioprine (AZA), tacrolimus), across categories of sex, race/ethnicity, socioeconomic status (SES), US region of residence, residence in a designated Health Professional Shortage Area (HPSA), and quartiles of pediatric rheumatologist number in state of residence. We tested for differences across categories using chi-squared and Fisher's exact tests, and applied the Cochrane Armitage test for trend.

## Results

Of the 254 pediatric lupus nephritis patients who developed ESRD, the mean age was 14.2 (± 2.4) years; 72% were female, 61% were African American and 19% were Hispanic. The mean time from first SLE claim to ESRD was 3.8 (± 2.1) years. A total of 46% had at least one renal biopsy preceding ESRD. More children in the lower quartiles of SES and the higher quartiles of rheumatologist number per state, received a biopsy (*P *trend < 0.05) (Table 1). Ninety-one percent of children were prescribed steroids at some time preceding ESRD, 63% were prescribed HCQ and 66% any other immunosuppressant, 50% of whom were prescribed MMF, 30% AZA and 14% CYC. We observed variation in prescribed steroids across region of residence, HCQ and immunosuppressant across race (more non-White patients prescribed both medications), and a greater proportion of patients prescribed HCQ in states with a higher number of rheumatologists per state.

**Table 1:** Proportion of pediatric patients with lupus nephritis-associated ESRD who received renal biopsies and medications prior to ESRD

		Renal biopsy		Corticosteroids		HCQ		Immunosuppressants	
		
		*n *(%)	*P *value^a^	*n *(%)	*P *value^a^	*n *(%)	*P *value^a^	*n *(%)	*P *value^a^
Total	254								
Sex									
Female	182	89 (49)	0.15	167 (92)	0.47	117 (64)	0.38	121 (66)	0.85
Male	72	28 (39)		64 (89)		42 (58)		47 (65)	
Race/ethnicity									
White	25	14 (56)	0.52	22 (88)	0.19	11 (44)	1 × 10^6^	13 (52)	0.02
Black	155	74 (48)		139 (90)		91 (59)		103 (66)	
Hispanic	48	21 (44)		47 (98)		44 (92)		34 (71)	
Asian	17	-		16 (94)		-		15 (88)	
Native	-	-		-		-		-	
SES group									
Quartile 1 - lowest	63	37 (59)	0.02^b^	60 (95)	0.21^b^	36 (57)	0.33^b^	42 (67)	0.95^b^
Quartile 2	63	27 (43)		56 (89)		41 (65)		42 (67)	
Quartile 3	63	29 (46)		58 (92)		39 (62)		42 (67)	
Quartile 4 - highest	64	23 (36)		56 (88)		43 (67)		42 (66)	
Region of residence									
Northeast	35	10 (29)	0.09	30 (86)	0.004	22 (63)	0.01	30 (58)	0.53
Midwest	52	26 (50)		43 (83)		23 (44)		23 (66)	
South	123	63 (51)		120 (98)		81 (66)		85 (69)	
West	44	18 (41)		38 (86)		33 (75)		30 (68)	
Residence in health professional shortage area									
Not HPSA	13	-	0.78	13 (100)	0.48	-	0.09	11 (85)	0.12
HPSA	240	110 (46)		217 (90)		151 (63)		157 (65)	
Pediatric rheumatologist per state									
Quartile 1 - lowest	63	38 (60)	0.0002^b^	59 (94)	0.24^b^	35 (56)	0.02^b^	43 (68)	0.50^b^
Quartile 2	64	34 (53)		62 (97)		37 (58)		46 (72)	
Quartile 3	72	30 (42)		63 (88)		46 (64)		42 (58)	
Quartile 4 - highest	55	15 (27)		47 (85)		41 (75)		37 (67)	

-, cell sizes under 11 have been suppressed in accordance with Centers for Medicare and Medicaid Services policy to protect privacy. ^a^*P *value for chi-square tests and Fisher's exact tests for small cell counts. ^b^*P *value for Cochran-Armitage Trend tests.

## Conclusion

We observed significant differences in the proportion of children who had received renal biopsies across categories of SES and rheumatologist number per state, as well as marked differences in medication prescribing across categories race, SES, regions of residence and rheumatologist number in state of residence.

